# Plasma protein GDF15 has a good predictive potential for the kidney complications of type 2 diabetes

**DOI:** 10.3389/fendo.2026.1758267

**Published:** 2026-05-11

**Authors:** Ming Hao, Houxing Li, Mengyu Xin, Jiatong Li, Rui Sun, Qian Liu, Yujie Zhang, Xinxin Shan, Yuting He, Borui Xu, Qiuyan Guo, Hongyu Kuang, Peng Wang

**Affiliations:** 1 Department of Endocrinology, the First Affiliated Hospital of Harbin Medical University, Harbin, China; 2 College of Bioinformatics Science and Technology, Harbin Medical University, Harbin, China; 3 Department of Gynecology, the First Affiliated Hospital of Harbin Medical University, Harbin, China

**Keywords:** complications, machine learning, metabolomics, proteomics, type 2 diabetes

## Abstract

**Introduction and aims:**

Complications of type 2 diabetes are a primary cause of public health challenges in the field of diabetes. The emergence of metabolomics and proteomics provides a direct perspective for revealing the mechanisms of metabolic diseases. Our research aims to explore the relationship between omics components and complications, as well as their clinical predictive performance.

**Materials and methods:**

This prospective study utilized data from the UK Biobank, including over 1,400 proteins and more than 280 metabolites, to analyze outcomes such as type 2 diabetes, microvascular complications, macrovascular complications, neurological complications, kidney complications, retinal complications, cardiovascular complications, peripheral vascular complications, metabolic disorder complications, and all-cause mortality. A total of 50,021 participants without type 2 diabetes were included in the analysis. The baseline time frame spanned from 2006 to 2010, with an average follow-up duration of 12.0 to 12.03 years. Researchers used LASSO Cox and LightGBM to search for new markers of complications, and employed SHAP methods to explain the contributions of these markers within the machine learning models. Subsequently, a comprehensive prediction model was established to reveal the potential of new markers for the early diagnosis of complications under nonlinear patterns, utilizing nine specific machine learning methods (CatBoost, LightGBM, Random Forest, XGBoost, logistic regression, multi-layer perceptron, single-layer neural network, Naive Bayes, and support vector machine).

**Results:**

GDF15 alone is more accurate than blood glucose and HbA1c in reflecting future kidney complications, especially in differentiating those who develop the disease within the next five years (GDF15 AUC=0.94, blood glucose AUC=0.68, HbA1c AUC=0.85). Within the framework of the comprehensive prediction model, the GDF15 model improved the accuracy of early screening for kidney complications compared with models constructed using traditional indicators (5-year Max AUC=0.92, 10-year Max AUC=0.88). In conclusion, both machine learning and statistical methods support the correlation between GDF15 and kidney complications, reflecting its robustness.

**Conclusions:**

The results highlight the association of GDF15 during the early asymptomatic stage of various complications, especially kidney complications, revealing the potential role of GDF15 at the molecular pathological level during disease progression. In distinguishing participants who developed complications after the baseline period, the comprehensive GDF15 model provides a method for the early warning of various complications, particularly kidney complications.

## 1 Introduction

Type 2 diabetes (T2D) is a form of diabetes characterized by elevated blood glucose levels, reduced insulin sensitivity, and a relative lack of insulin. In 2024, it is estimated that 589 million adults aged 20–79 were living with diabetes. Over 3.4 million people aged 20–79 died from diabetes-related causes. The total number of people living with diabetes is projected to reach 853 million by 2050 (1). Global data indicates that approximately 2 million deaths annually are attributable to diabetes and its complications. It is commonly assumed that complications are the underlying cause of death (2, 3). Early intervention can significantly delay the progression of complications or reduce their impact on patients, ultimately improving their quality of life. During the prediabetic stage, HbA1c is affected by pathological factors such as iron deficiency anemia, splenomegaly, severe hyperbilirubinemia and severe hypertriglyceridemia, which may lead to inaccurate elevation of its level (4–7). The oral glucose tolerance test and the level of glycated albumin can also be affected by non-diabetic factors such as diet, stress levels, liver diseases, and inflammation (8, 9). Therefore, developing more specific and sensitive indicators for identifying early diabetic complications is an important issue that urgently needs to be addressed.

At present, most studies on diabetic complications based on proteomics and metabolomics employ cross-sectional designs, and their limitation lies in redirection. Due to the heterogeneity of diseases, the complexity of omics technologies, as well as various practical and ethical considerations, large-scale prospective longitudinal cohorts specifically targeting kidney damage caused by diabetes are still relatively scarce (10). The UK Biobank (UKB) project offers researchers plasma proteomics and metabolomics data from over 50,000 individuals, while also recording a wealth of disease follow-up outcomes and demographic traits. This provides researchers with high-quality data resources. Several recent representative studies using UK Biobank data have demonstrated the potential of protein biomarkers in predicting the risk of T2D (11–13). Based on these data, we conducted an original study with the aim of identifying valuable markers that could indicate the asymptomatic early stage of complications associated with T2D.

We utilized the plasma proteomics and metabolomics data of 50,021 individuals from the UKB, and employed multivariate statistical analysis and machine learning methods to explore shared and specific markers for different complications, including macrovascular complications (MaVC), microvascular complications (MiVC), cardiovascular complications (DCVD), kidney complications (DKD), neurological complications (DN), peripheral vascular complications (DPVD), retinal complications (DR), and metabolic disorder complications (MetD). Eventually, these key markers were used to construct a comprehensive complication prediction model.

This study aims to reveal the potential pathological mechanisms of proteins and metabolites in diabetes complications, providing new tools for early warning and risk stratification of complications.

## 2 Methods

### 2.1 Study design and cohort

**Figure 1:**
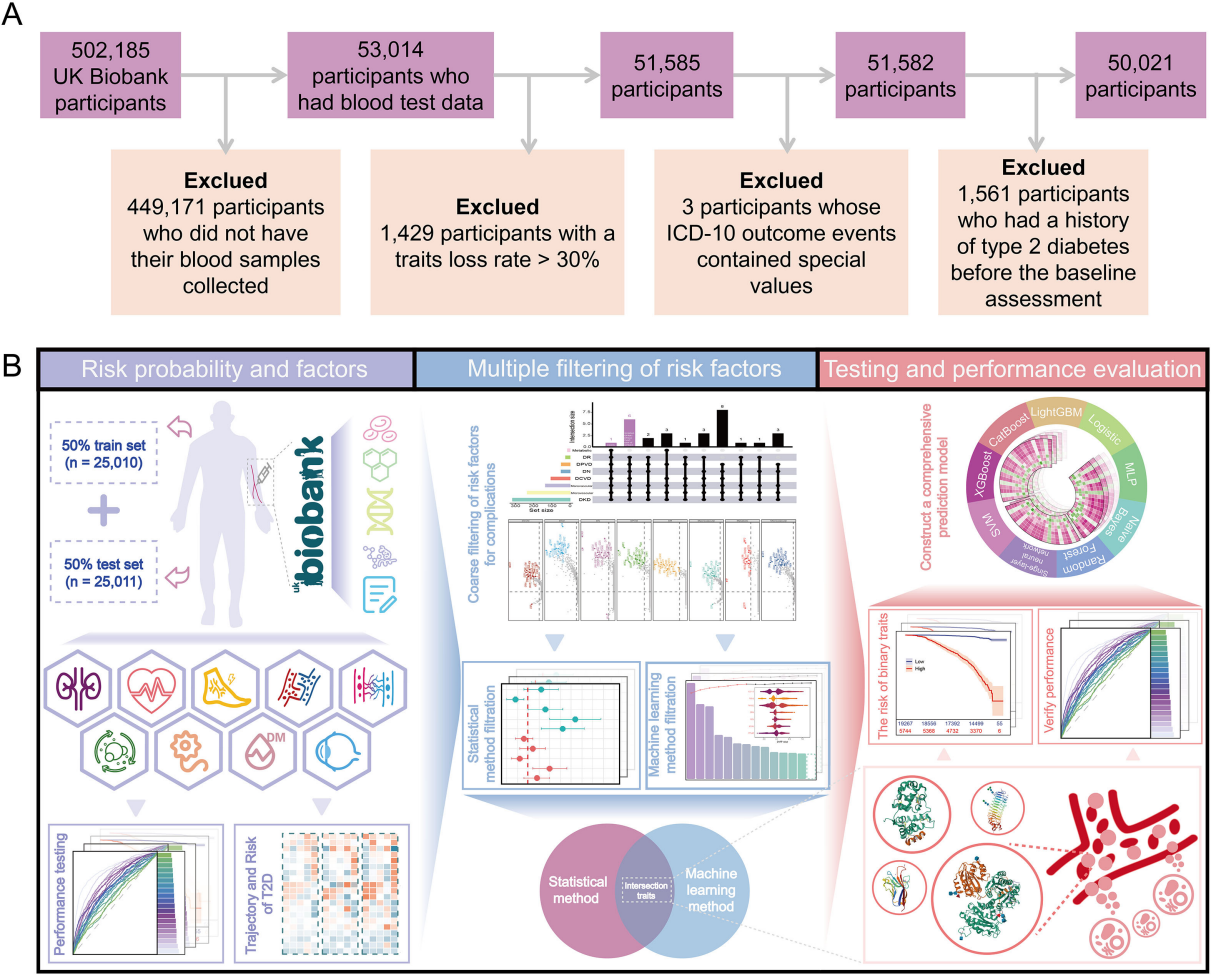
Research Design. **(A)** Participant exclusion process. **(B)** Research process.

The UKB is a large population-based prospective cohort. From 2006 to 2010, over 500,000 participants aged 39 to 70 years from 22 assessment centers in England, Scotland, and Wales participated. Approximately 50,000 of them agreed to have their blood samples collected (14). This study included 50,021 participants who met the following criteria: 1) agreed to have their blood samples collected; 2) had a trait loss rate < 30%; 3) the ICD-10 code for the outcome event did not contain a special value (Field ID = 130708 Notes); 4) had no history of T2D before the baseline period. **Figures 1A, B** shows show the flowchart of the research.

### 2.2 Participant traits

The traits used can be classified primarily as demographic and clinical traits, proteomic traits, metabolomic traits, blood antigens, and a small number of blood cell traits (**Supplementary Table 2**). A total of 53,014 participants agreed to have their blood samples collected. These samples were sent to the Olink Analytical Service Centre in Sweden for protein component testing. Proteomics data were subjected to strict quality control (Resource ID = 4658), and a total of 1,463 unique proteins were detected. The traits and clinical data of the participants were anonymized for the researchers. After analyzing the proteomics data, UKB processed the data using a normalization method to obtain NPX values. The details of the collection, detection, and quality control of the proteomics data have been disclosed in previous publications (Category ID = 1839), so proteomics data are assumed to follow a normal distribution (15–17). Metabolites and racial traits have been appropriately simplified; for details, please refer to the Supplementary Information. Traits with a missing rate of over 50% were excluded, and missing values were imputed using the mice R package.

### 2.3 Outcome events

The codes for defining diseases, the relationships between diseases, and the abbreviations used are included in **Supplementary Table 5**. Only participants with a confirmed record of T2D who are subsequently recorded as having related complications will be classified as patients with T2D complications. Those without a T2D diagnosis record, but with complication records, are not included in this category. Following the determination of the T2D onset date, the codes of complications that occurred subsequently were utilized to determine the occurrence dates of those complications. The presence of any one of these complications is defined as ‘Total Complication’ (**Supplementary Table 5**). The data processing method for survival status can be found in the Supplementary Information. Following processing, the baseline date, the T2D date, the complication date, and the all-cause death date were obtained. For some participants, these four dates overlap. To distinguish between different date nodes, one day was added to the overlapping dates that occur later in the trajectory.

### 2.4 Statistical analysis

The participant cohort was randomly divided into train (n=25,010) and test (n=25,011) sets, and the differences in trait distributions between the two datasets were examined (**Supplementary Table 3**).

A multi-state model was employed to analyze the risk associations of multiple traits with T2D trajectories, and these traits were also used to estimate risk probabilities for future T2D progression stages (18) (see Supplementary Information).

The multi-state model investigated the risk factors associated with T2D and total complications. Subsequently, further analysis was conducted to determine which traits influence specific types of complications in train cohort. The precise positive and negative sample sizes are recorded in **Supplementary Table 1**. The cohort was divided into the complication population and the non-complication population. The Cox proportional hazards regression model was utilized to ascertain the association between traits and complications (**Supplementary Table 8**). In addition, Panel 1 was added to adjust the model (details of Panels 1 and 2 are in the Supplementary Information) (**Supplementary Tables 18, 19**). The Bonferroni method was employed to correct the p-values for multiple hypothesis testing (**Supplementary Table 8**).

The subsequent section discusses the importance of proteins identified through machine learning modelling. Traits that demonstrated significance following Bonferroni correction were integrated into a LightGBM model, which facilitated binary classification predictions for complications and non-complications. The binary outcome was divided according to the onset time frame: within 5 years, 10 years, and all incident years (the longest period being approximately 17 years). Note that no analysis was conducted when the number of positive cases within a time frame was fewer than 20. Initially, the corrected traits were entered into a preliminary LightGBM model, and the importance scores of these traits were derived. Subsequently, traits were added sequentially in descending order of importance to train a LightGBM model. The model AUC values were calculated for each added trait. We used a method of fitting segmented regression curves to identify breakpoints and key traits in AUC elevation (19). We then utilized a method capable of calculating SHAP values for tree-based models in polynomial time to ascertain the contribution of each trait to the predicted outcomes and the direction of the effect (20).

To enhance the perceived credibility of the key traits, we used LASSO-Cox regression to process the train cohort (21). The traits obtained were then utilized to construct a multivariate Cox proportional hazards regression model.

Receiver operating characteristic (ROC) analysis and Kaplan-Meier (KM) survival curves were used to explore the performance of the traits (see Supplementary Information).

After identifying the intersecting traits, we aimed to utilize a small subset of these traits to enhance clinical predictive performance and explore their potential for clinical application. We selected several methods, including tree-based models (CatBoost, LightGBM, Random Forest, and XGBoost), logistic regression, artificial neural networks (multi-layer perceptron and single-layer neural networks), Naive Bayes, and support vector machines (see Supplementary Information). These include both machine learning models adept at handling nonlinear relationships and statistical models providing clear interpretability. These methods were chosen because they are well-established and demonstrate reliable performance in clinical predictive modeling (22–30).

A two-tailed P < 0.05 was considered statistically significant. The tools used for data processing and visualization are listed in **Supplementary Table 20**.

## 3 Results

### 3.1 Participants’ traits

The prospective study cohort included 50,021 individuals from the UKB. The population was segmented into a train subcohort and a test subcohort. The baseline time frame covered the period from 2006 to 2010. The mean lengths of follow-up were 12.00 ± 3.20 and 12.03 ± 3.16 years, respectively. The mean age of the train and test sets were 56.7 ± 8.19 and 56.7 ± 8.25 years; HbA1c was 35.8 ± 5.89 mmol/mol and 35.9 ± 6.24 mmol/mol; BMI was 27.4 ± 4.68 and 27.3 ± 4.69; and glucose (biochemistry) was 5.08 ± 1.10 mmol/L and 5.09 ± 1.12 mmol/L. The number of females was 13,558 (54.2%) and 13,694 (54.8%), respectively. The number of individuals documented to have developed T2D during follow-up was 1,775 (7.1%) and 1,792 (7.2%). The number of individuals who experienced any of the complications was 798 (44.96%) and 800 (44.64%) of patients with T2D. Specifically, the most common complications were DCVD and DKD. The mortality observed in the two subcohorts was 2,594 (10.4%) and 2,476 (9.9%) (**Supplementary Table 1**). The findings revealed that 93 traits had p-values < 0.05, accounting for 5.1% of all 1,824 traits. There was no difference in the distribution of the main traits between the two subcohorts (**Supplementary Table 3**). Therefore, the utilization of these two subcohorts was maintained.

### 3.2 Proteomics estimates the risk of T2D and its complications

A multi-state model was utilized to characterize participants’ full-flow T2D disease progression. CCL22 (HR [95% CI] = 1.78 [1.52-2.07]), ADS (1.77 [1.46-2.15]), CXCL10 (1.79 [1.53-2.09]), GRPEL1 (2.16 [1.84-2.54]), CD164 (1.54 [1.32-1.79]), CEISLDL (1.22 [1.03-1.45]), and CARHSP1 (1.2 [1.05-1.38]) increased the risk of T2D. In a similar vein, a statistically significant effect of ADS (1.51 [1.15-1.97]) was observed at the Q4 level of Transition 3 in comparison to the Q1 level (**Supplementary Figure 1B**).

Taking CXCL10 as an example, 16 years after the baseline period, the probability that Q4 patients with CXCL10 were still in a healthy state was 65.66%, and the probabilities of T2D, total complications, and death were 2.45%, 5.10%, and 26.79%, respectively. The risk of T2D in Q4 was 1.37 times that of Q1, and the risk of total complications was 1.49 times higher (**Supplementary Figure 2**).

### 3.3 GDF15 has a strong correlation with DKD

**Figure 2:**
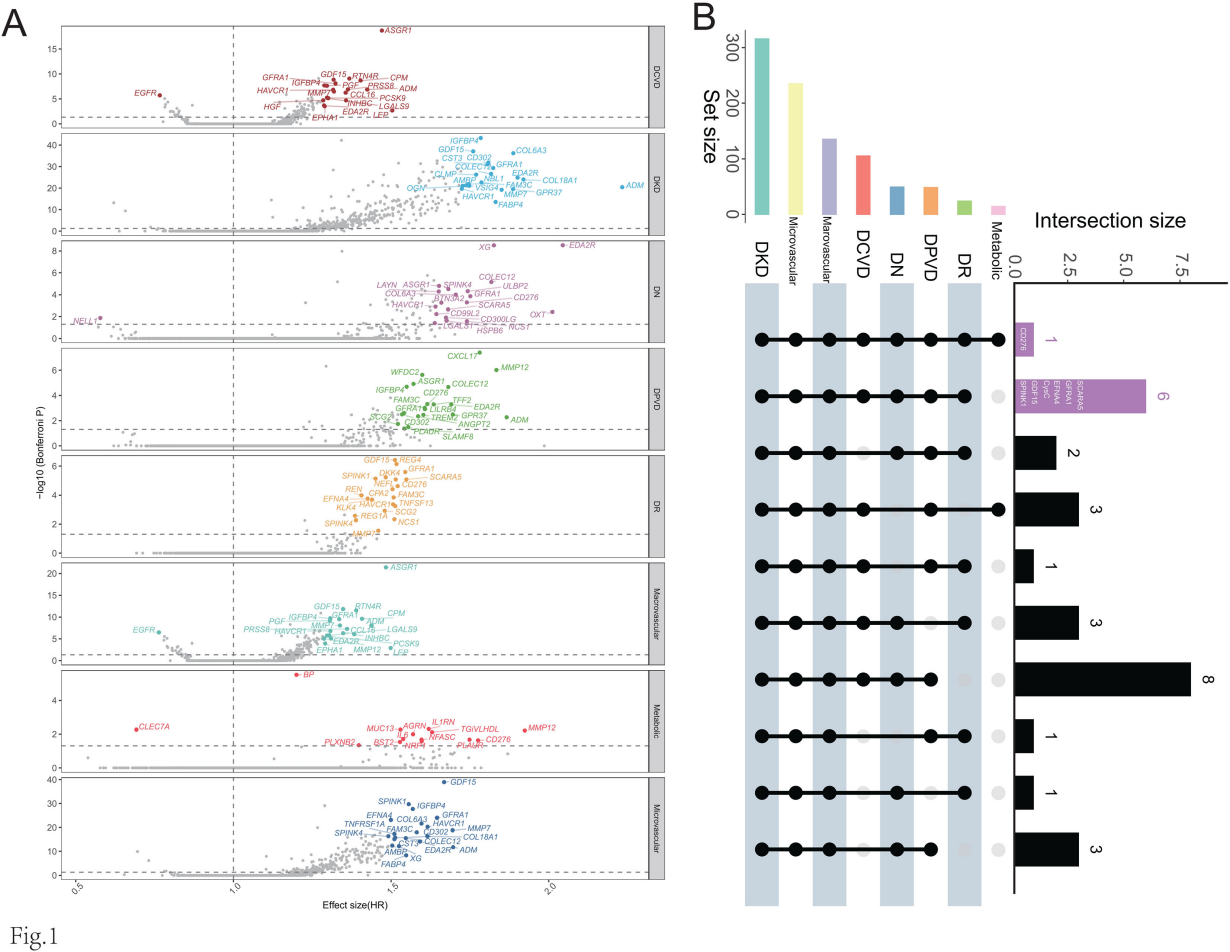
Complication-associated proteins and metabolites. **(A)** Volcano plot of complications and traits. **(B)** Intersection of complication-associated traits.

The multi-state model investigated the risk factors associated with T2D and total complications. Subsequently, a more detailed analysis was conducted to understand how proteins and metabolites affect specific complications. After adjusting for Panel 1 and applying the Bonferroni correction, we found that the specific complications had 235, 135, 49, 316, 24, 105, 48, and 14 significant traits, respectively (**Figure 2A** and **Supplementary Table 8**). Among these complications, DKD had the highest number of associated traits, totaling 316. High HR values were also more prevalent in DKD, and the minimum p-value for the significant traits was notably lower (**Figure 2A** and **Supplementary Table 8**). These findings suggest that blood markers have the potential to reflect the progression of DKD. Notably, the CD276 protein was shown to have a significant effect on all eight types of complications. Except for MetD, GDF15 has significant risk effects on all other complications (**Figure 2B**). The adjusted p-value of GDF15 for DKD is 8.52×10^-38^, and the HR [95% CI] is 1.76 [1.62 - 1.91]. The adjusted p-value of GDF15 is only lower than those of IGFBP4 and CysC (4.66×10^-44^, 5.89×10^-43^). The HR value of GDF15 is slightly lower than that of the top-ranked ADM (HR [95% CI] = 2.23 [1.91 - 2.60]) (**Supplementary Table 8**). Overall, GDF15 showed a strong association with DKD in the initial analysis.

### 3.4 The independent association between GDF15 and DKD is supported by interpretable machine learning methods and statistical analysis

**Figure 3:**
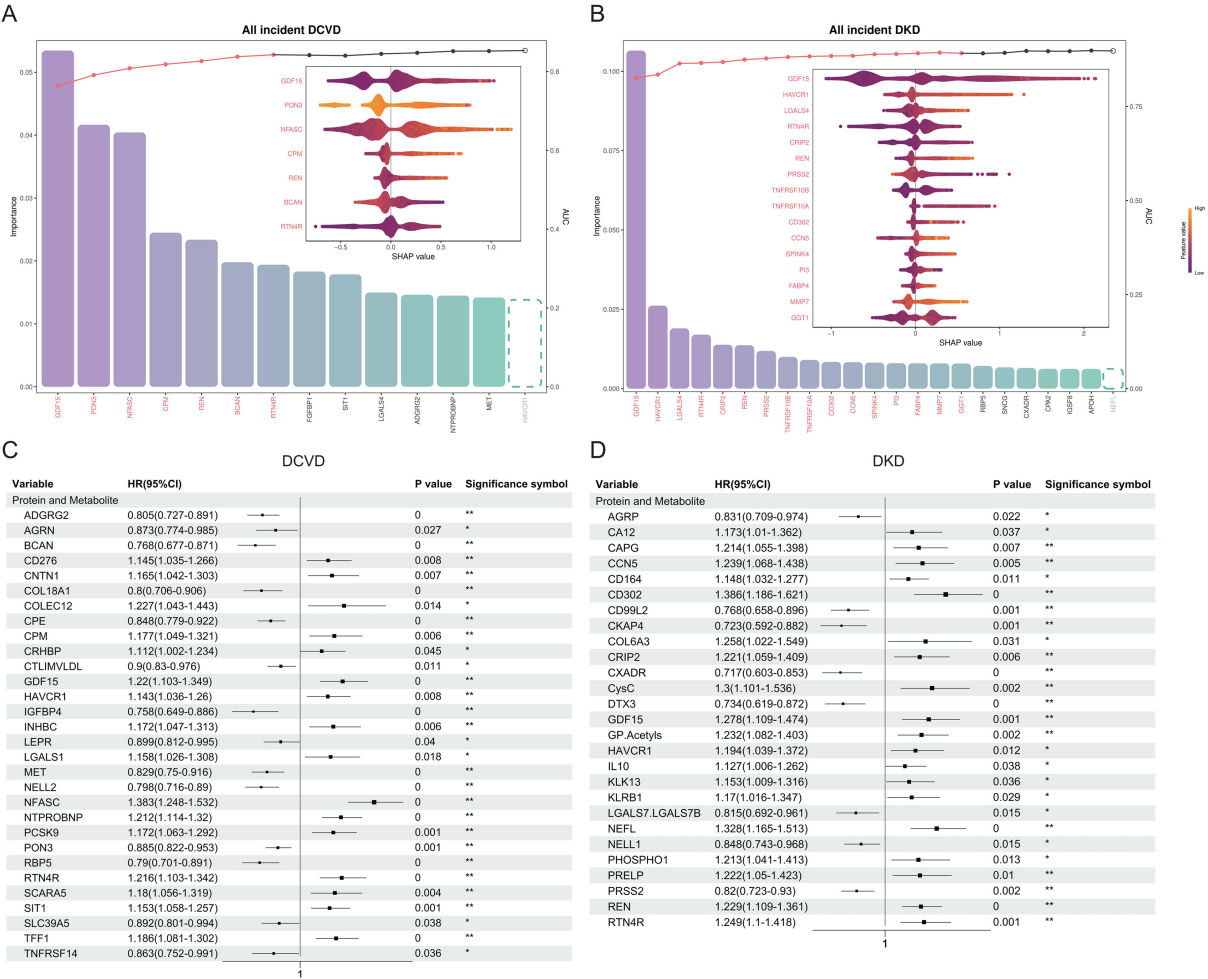
Complication-related traits further screened out by the two methods. **(A, B)** Key traits of DCVD and DKD events within all incident period screened by machine learning methods. The bar chart displays the traits that have been assigned the highest importance scores, while the SHAP swarm plot illustrates the contribution of key traits to the prediction of complications. The model AUC changes brought about by the sequential addition of traits are presented in the form of scatter plots and broken lines. **(C, D)** Key traits of DCVD and DKD screened by statistical methods. “*” indicates p-value < 0.05, “**” indicates p-value < 0.01, “0” indicates p-value < 0.001.

Importance analysis of the LightGBM model’s AUC values shows that two key proteins (RTN4R and GDF15) are present in DCVD, and four proteins (REN, CDH15, SPINK4, and HAVCR1) for DR, five for MaVC (GDF15, RTN4R, BCAN, NFASC, and PON3), two for MiVC (GDF15 and REN), five for DPVD (TFF2, REN, NTF3, KLK4, and GDF15), two for DKD (LGALS4 and GDF15), three for MetD (MMP12, CLE7A, and PLXNB2), and six for DN (GDF15, HAVCR1, OXT, CDH15, PTPRN2, PTPNB2) (**Figures 3A, B** and **Supplementary Figure 3**, **4**). In addition to DR and MetD, GDF15 has been identified as a key trait for all types of complications. It was been observed that REN is a prevalent key trait of DR, MiVC, and DPVD. CDH15, SPINK4, and HAVCR1 are also common key traits of DR and DN. In addition, GDF15 is the only common key trait of DCVD, MaVC, MiVC, and DKD within a 5-year period. Within 10 years, GDF15 was identified as a common key trait, except for MetD and DR. Within all incident periods, GDF15 was identified as a common key trait, except for MetD. REN has been identified as a common key trait, except for MetD and DN. SPINK4 and HAVCR1 have been identified as the common key traits of DR, MiVC, DN, and DKD (**Supplementary Table 9**)-(**Supplementary Table 16**). In the SHAP swarm plot, GDF15 demonstrates the widest range for DKD and MiVC within all incident periods (**Figure 3B** and **Supplementary Figure 4F**), which indicates that it exhibits excellent predictive performance for these diseases. Concurrently, the alteration in the scatter color of GDF15 suggests that it may have a risk-increasing effect on these complications (**Figure 3B** and **Supplementary Figure 4F**).

To provide further confirmation, using the LASSO Cox regression statistical method was used to analyze traits related to complications (**Figures 3C, D** and **Supplementary Figure 5**, **6**). Except for MetD and DPVD, GDF15 and HAVCR1 demonstrated a significant positive association with other complications (**Supplementary Figures 5A–C** and **6A, B, D**). REN was been found to be significantly associated with DKD, DPVD, MiVC, and DR (**Supplementary Figures 5B, D** and **6AD**), which corresponds to the results identified by the machine learning method (**Supplementary Figure 3**DF and 4D-J]. CDH15 is associated with DN, MiVC, and DR (**Supplementary Figure 5C** and **6AD**], and SPINK4 is associated with DN, MaVC, and DR (**Supplementary Figure 5C** and **6B, D**). Both statistical and machine learning methods identify BCAN and PON3 as protective factors for DCVD and MaVC (**Supplementary Figures 3A,C, E**, **4A-C**, **5A**, and **6B**). Under both two methods, NELL1 is also a protective factor for DKD (**Supplementary Figure 3BD** and **5B**).

### 3.5 The discriminatory power of GDF15 for DKD patients is superior to that of traditional indicators

**Figure 4:**
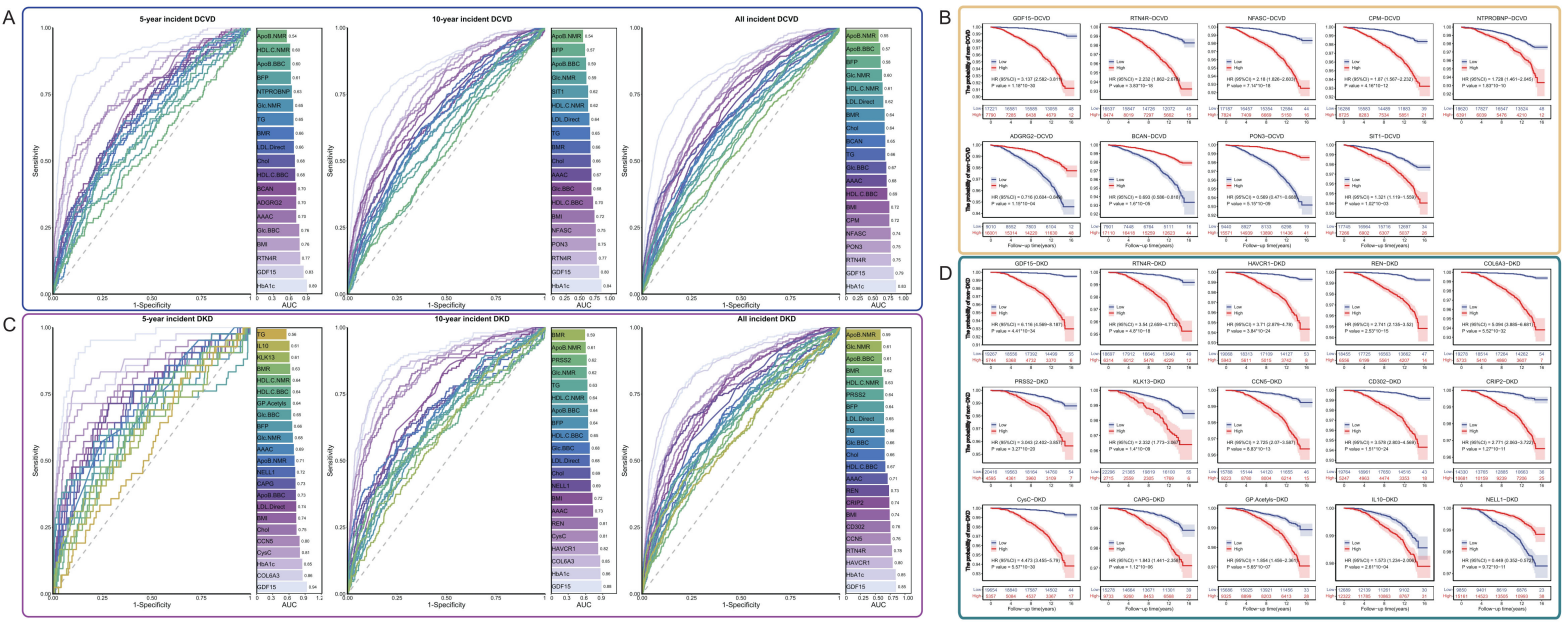
Predictive performance of intersection traits. **(A, C)**. ROC analysis comparing the predictive accuracy of DCVD- and DKD-specific traits versus clinical traits for future complication events. AUC values are displayed as bar charts. ROC curves correspond to AUC values by color. **(B, D)**. KM survival curves for DCVD and DKD in high- and low-groups of specific traits. HR values and p-values for both groups were calculated using Cox analysis with binary-classified traits.

Among common clinical indicators, HbA1c has been shown to be a strong predictor of complications, with AUC values consistently exceeding 0.83 (**Figures 4A, C** and **Supplementary Figure 7**). Beyond DPVD, DR within 10 years, and MetD, GDF15 demonstrated the most optimal predictive performance for other complications among the key intersection traits (**Figures 4A, C** and **Supplementary Figure 7**). The AUC values of GDF15 for DKD, MiVC within 5 years, and DN within 10 years exceed 0.85, with the AUC for DKD within 5 years reaching 0.94 (**Figure 4C** and **Supplementary Figures 7D, G**). For DPVD, DR within 10 years, and MetD, the key intersection traits demonstrating the highest performance are REN (AUC = 0.81), ASGR1 (0.79), HAVCR1 (0.76), and PLXNB2 (0.79) (**Supplementary Figures 7I, J, K, M**). A comparison of the AUC values between key intersection traits and common clinical indicators revealed that the AUC of GDF15 in predicting DKD was higher than that of HbA1c (**Figure 4C**). The predictive performance of GDF15 for DKD within a 5-year timeframe was most prominent, yielding an AUC of 0.94 compared to 0.85 for HbA1c (**Figure 4C**). In most other cases, the predictive performance of GDF15 was second to that of HbA1c. Furthermore, the maximum short-term predicted AUC values for all complications were higher than the maximum long-term values.

Furthermore, survival analysis yielded largely consistent results. With the exception of ACP5-DR and OXT-DN, the remaining 70 trait-complication pairs all exhibited significant associations (**Supplementary Table 17**). Except for DPVD and MetD, the HR of GDF15 for all other complications was greater than 3 (**Figures 4B, D** and **Supplementary Figures 8A, B, C, E**). The effects on DKD (HR [95% CI] = 6.116 [4.569-8.187]), MiVC (4.574 [3.51-5.96]), and DN (4.082 [2.413-6.906]) were the most pronounced (**Figure 4D** and **Supplementary Figures 8C, E**). RTN4R (2.232 [1.862-2.676]) and NFASC (2.18 [1.826-2.603]) also demonstrated significantly increased risks for DCVD (**Figure 4B**). The effects of COL6A3 (5.094 [3.885-6.681]), CysC (4.473 [3.455-5.79]), HAVCR1 (3.71 [2.879-4.78]), CD302 (3.578 [2.803-4.569]), and RTN4R (3.54 [2.659-4.713]) on DKD were also very prominent (**Figure 4D**). A significant reduction in the risk of complications was observed for ADGRG2-DCVD, BCAN-DCVD, PON3-DCVD, NELL1-DKD, PON3-MaVC, ADGRG2-MaVC, and BCAN-MaVC (**Figures 4B, D** and **Supplementary Figures 8B, C**).

### 3.6 The model constructed using the new markers has improved the efficiency of early screening for complications

**Figure 5:**
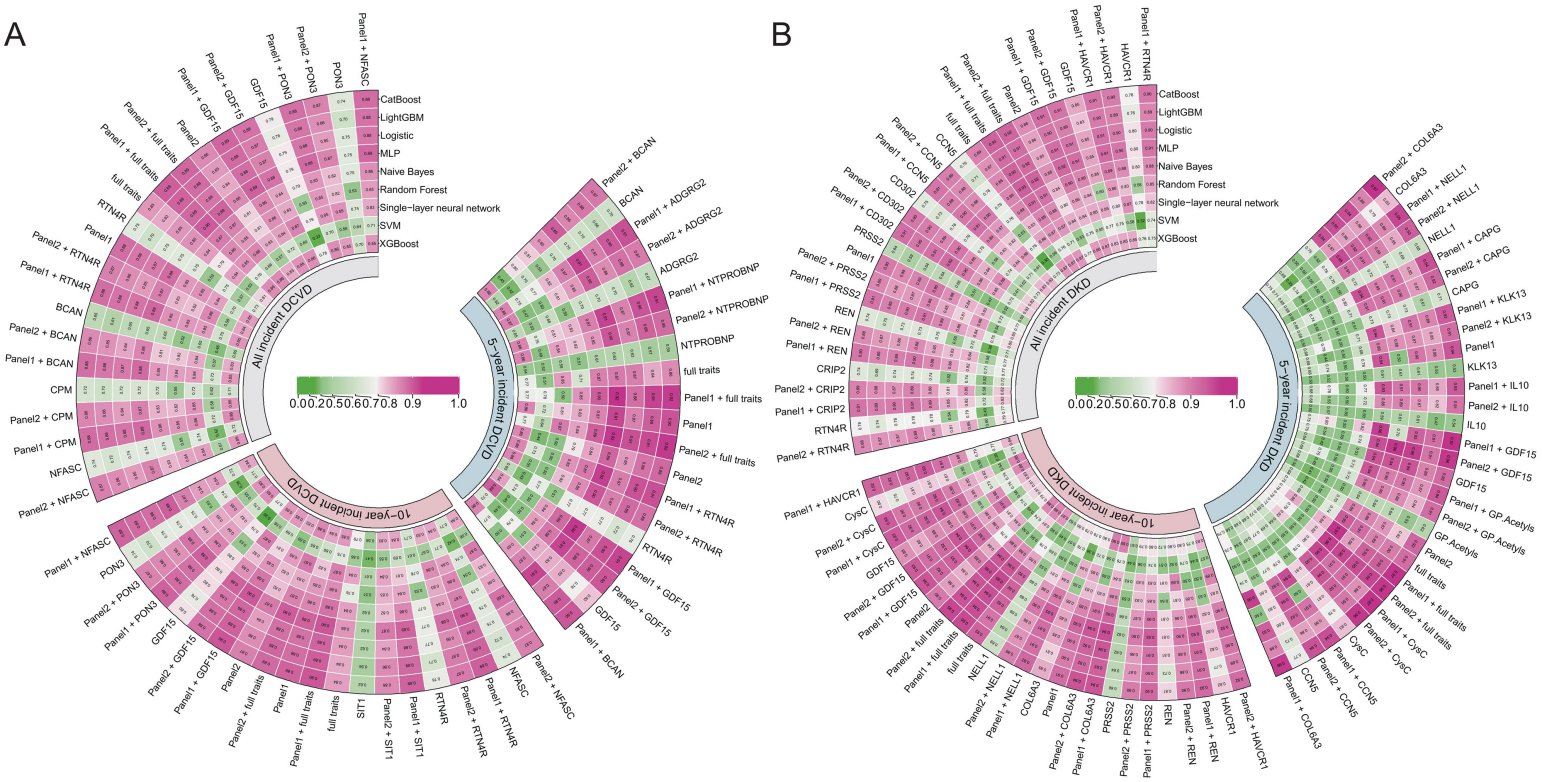
Performance evaluation of the comprehensive model constructed by intersection traits. **(A, B)** AUC values calculated by the comprehensive model for DCVD and DKD at different disease onset stages. Squared numbers indicate AUC values.

In predicting DCVD events, the comprehensive model constructed using GDF15 alone has been shown to have a higher prediction accuracy for complication events than any other individual intersection trait. The maximum AUC values of the 5-year, 10-year, and all incident models are 0.83, 0.80, and 0.79, respectively. When predicting DCVD within all incident period, the predictive performance of full traits (maximum AUC = 0.85) exhibited a substantial enhancement in comparison to GDF15 alone (maximum AUC = 0.79). In addition, the performance of full traits + Panel2 was enhanced across all time frames. Specifically, the maximum AUC is higher than that of the model incorporating full traits or Panel2 alone (**Figure 5A**).

In predicting DKD events, the prediction accuracy of GDF15 alone is higher than that of any other single intersection trait. The maximum AUC values of the 5-year, 10-year, and 17-year models are 0.94, 0.80, and 0.79, respectively. It is noteworthy that, in predicting DKD within a 5-year time frame, the accuracy of GDF15 alone (0.94) surpasses that of the full traits (0.93). It is analogous to the prediction accuracy of the Panels (0.94 and 0.92). Moreover, the GDF15 + Panel1 model has a maximum AUC value of 0.97, which represents a certain improvement compared to GDF15 (0.94) and Panel1 (0.94) alone. Particularly, the maximum AUC values of CysC + Panel1 and COL6A3 + Panel2 both reached 0.97, and the maximum AUC of both GDF15 + Panel2 and CysC + Panel2 is 0.96. In comparison with Panel2 (0.92), this is a significant performance improvement. Clearly, the full traits + Panel2 model shows higher accuracy across all time frames compared to the Panel2 model. The findings indicate that GDF15 and full traits alone possess the capacity for elevated clinical predictive accuracy for DKD. Conversely, the combination of common clinical indicators with GDF15 or full traits has been demonstrated to yield performance that exceeds that of common clinical indicators (**Figure 5B**).

When considering the predictions for DR and DN, GDF15 remains the single best-performing intersection trait. The respective full traits performed better than the individual intersection traits (**Supplementary Figures 9A–D**). When predicting DPVD within all incident period, the maximum AUC of the full traits was 0.87, which was higher than that for the single intersection trait. The maximum AUC of the full traits + Panel 2 was 0.93, showing an improvement over Panel 2 (0.90) (**Supplementary Figure 9E**). When predicting MetD within all incident period, the maximum AUC of the full traits was 0.80, which was higher than that of the single intersection trait (**Supplementary Figure 9F**).

## 4 Discussion and conclusions

Our research identified plasma proteomic and metabolomic components associated with T2D and its complications. We found that the GDF15 protein is a key protein for various complications of T2D. Except for DPVD and MetD, the predictive accuracy of GDF15 exceeds that of other proteomic and metabolomic components. When constructing a comprehensive model for predicting DKD, GDF15-based models can provide a sufficiently high level of accuracy. Its accuracy exceeds or is equivalent to that of models constructed using clinical indicators such as HbA1c. When GDF15 is combined with common clinical indicators, its effectiveness in predicting outcomes is significantly improved. In the context of DKD, we identified several additional key proteins (COL6A3, CysC, and HAVCR1). The additional key proteins for DCVD are RTN4R, NFASC, and PON3. The additional key protein for DN is HAVCR1. The extra key proteins in DR are HAVCR1, SPINK4, and REN. The key proteins for DPVD are REN and ASGR1. The PLXNB2 protein in MetD is worthy of consideration.

Previous studies have indicated that GDF15 is a signal for kidney damage in DKD, rather than a causative factor (31, 32). Mazagova et al. discovered that GDF15 in kidney tissue was strongly upregulated before the development of DKD (32). Li et al. conducted a study comparing the serum concentrations of GDF15 in patients in the normal proteinuria phase, early stage of DKD, and overt DKD phase. They found that in the early and overt stages of DKD, the levels of GDF15 had showed a detectable and significant increase; as DKD persisted and progressed, the increase in GDF15 became more pronounced (33). Mazagova et al. also found that at 18 weeks of age, T2D mice with the GDF15 gene knocked out exhibited more severe renal tubular damage than T2D mice without the gene deletion (32). This direct evidence clearly demonstrates the significant role of GDF15, namely its protective effect on the kidneys in the context of diabetes. Mechanistically, GDF15 inhibits the AGE/RAGE axis and downstream inflammatory factors, thereby inhibiting the activation of the TLR4/MyD88 pathway and preventing the activation of IKK. On the other hand, GDF15 inhibits the expression of NEDD4L, which inhibits the ubiquitination degradation and phosphorylation activation of IKK, ultimately leading to the inhibition of the nuclear translocation of NF-κB (34–36). These pieces of evidence indicate that GDF15 is a tissue-protective repair factor for kidney damage caused by high glucose levels, and NF-κB is the downstream core node that mediates this mechanism. Mazagova et al. pointed out that, “Although the role of GDF15 as a biomarker and protective factor may appear paradoxal, these studies in GDF15 knockout mice demonstrate the protective properties of GDF15 in the kidney and heart, respectively.” Overall, we hypothesize that high blood sugar causes damage to the kidney tissues, and these tissues upregulate the expression of GDF15 to repair the damage. This not only explains the protective effect of GDF15 but also explains the marker characteristics of GDF15 relative to the severity of kidney damage. The extensive secretion of GDF15 from damaged tissues also explains the significant increase we observed in its plasma levels. The results of this study indicate that plasma GDF15 performs well in predicting DKD over the next five years. The aforementioned research also detected abnormal GDF15 levels before and during the early stages of DKD symptoms (32, 33). Based on these evidences, it is concluded that GDF15 has extremely high sensitivity in reflecting kidney damage. Our results provide a specific reference time window for the early identification of DKD. In addition, our study covered over 1,400 types of plasma proteins. Among them, only GDF15 demonstrated extremely high predictive ability for DKD, proving that it has sufficient specificity for diagnosing DKD.

Observational studies across different ethnic groups and sample sizes have consistently shown that circulating levels of GDF15 are an independent predictor of DCVD in T2D (37, 38). GDF15 is positively correlated with cardiovascular damage and shows consistent upregulation during cardiovascular diseases, such as pressure overload, heart failure, ischemia-reperfusion injury, and atherosclerosis (39, 40). However, the effect of GDF15 on atherosclerosis is characterized by both protective and risk effects, while it shows clear protective effects on cardiovascular diseases such as heart failure and ischemia-reperfusion injury (41). Tian et al. suggest that the contradictory effects of GDF15 on atherosclerosis may be due to variations in experimental conditions and the lack of a systematic assessment of the severity of atherosclerotic lesions throughout the vascular system (41). After myocardial infarction, damaged cardiac muscle cells release GDF15. This substance interferes with the signal pathways activated by leukocyte integrins, directly inhibiting the recruitment of inflammatory cells, including neutrophils and monocytes/macrophages, thereby achieving a core protective effect by suppressing excessive inflammation and preventing cardiac rupture (42). Zhang et al. discovered that GDF15 protects against diabetes-induced endothelial dysfunction and oxidative stress damage by activating AMPK (43). Endothelial dysfunction is one of the earliest pathogenic events in diabetes-related vascular diseases (44). Our results indicate that GDF15 demonstrates already demonstrated significant predictive ability before the onset of DCVD, which may be attributed to its protective effect on endothelial dysfunction. This also indicates that GDF15 has the potential to diagnose early pathological changes in DCVD. Chan et al. discovered that GDF15, in the context of diabetes, exerts an indirect anti-inflammatory effect in cardiomyopathy, and this effect is not dependent on weight loss. This indirect effect is evidenced by the fact that using GDF15 to directly treat adult mouse cardiomyocytes and differentiated THP-1 human macrophages failed to alleviate inflammation induced by lipopolysaccharide (45). Existing evidence indicates that in the context of diabetes, GDF15 may be a repair factor released after cardiovascular damage. Chan et al. pointed out that during the process of high-sugar-induced cardiovascular damage, GDF15 exhibits anti-inflammatory effects, but the specific mechanism remains unclear, especially because the expression of its typical receptor, GFRAL, is highly localized in the posterior part of the central nervous system (45). This contradiction is also key to explaining the role of GDF15 in other diabetic complications. The strong correlation we discovered between circulating GDF15 and DCVD may provide new evidence to explain this contradiction; specifically, GDF15 is released from damaged cells in an endocrine form and transmits tissue repair signals by binding to the GFRAL receptor located in the posterior brain.

Furthermore, studies have shown that HAVCR1 is overexpressed in DKD, especially in damaged proximal renal tubular cells. It is involved in various pathways such as immunity, inflammation, apoptosis, and matrix remodeling, which can trigger DKD. HAVCR1 has been regarded as an early non-invasive biomarker and may be a potential mechanism for promoting renal tubular damage (46–48).

The advantage of this study is the implementation of multiple machine learning methods for the analysis of over 1,400 types of proteomics data and over 280 types of metabolomics data from a large-scale prospective cohort involving over 50,000 people. This approach enhances the clinical applicability of the findings through the utilization of SHAP technology.

However, it is important to note that this study has some limitations. The primary concern pertains to the absence of result validation in independent prospective cohorts. Secondly, while the current largest-scale plasma proteomics atlas has captured over 2,900 proteins (49), the dataset under consideration does not include all measurable proteomic components due to the inherent limitations of the UKB collection. Furthermore, the metabolomics data only contains over 270 metabolites.

Overall, these plasma components provide a basis for improving the clinical prediction performance of T2D complications and are helpful for the further efficient and specific prediction and diagnosis of various complications. Additionally, the high specificity and sensitivity of GDF15 for DKD and DCVD provide a basis for early warning and risk stratification. Based on our results and existing evidence, using circulating GDF15 as a rapid and non-invasive detection method is feasible. For future research, resolving the contradiction between the secretion of GDF15 by damaged tissues and the distribution of GFRAL receptors is key to improving the understanding of its pathological mechanism. For clinical practice, verifying the diagnostic performance of GDF15 in larger-scale, geographically and ethnically diverse populations with different types of diabetes and disease courses, confirming its universality, and establishing reliable clinical cutoff values will be an urgent priority.

## Data Availability

Publicly available datasets were analyzed in this study. This data can be found here: UK BioBank application number 102952. All software used in this study is publicly available. The code used in this study can be accessed at https://github.com/look-bef-u-leap/T2DComplication.
