# Supplementary material for: Plasma protein GDF15 has a good predictive potential for the kidney complications of type 2 diabetes
Source: Front Endocrinol (Lausanne). 2026 May 11;17:1758267. doi: 10.3389/fendo.2026.1758267 (PMC13199011; doi:10.3389/fendo.2026.1758267)
Supplement: Supplementary file 1 [file DataSheet1.pdf]

Supplementary Information

Plasma protein GDF15 has a good predictive potential for the kidney complications of type 2 diabetes

Ming Hao[†,1], Houxing Li[†,2], Mengyu Xin[†,2], Jiatong Li[2], Rui Sun[2], Qian Liu[2], Yujie Zhang[2], Xinxin Shan[2], Yuting He[2], Borui Xu[2], Qiuyan Guo[*,3], Hongyu Kuang[*,1,] Peng Wang[*,2]

1 Department of Endocrinology, the First Affiliated Hospital of Harbin Medical University, Harbin 150081, China

2 College of Bioinformatics Science and Technology, Harbin Medical University, Harbin 150081, China

3 Department of Gynecology, the First Affiliated Hospital of Harbin Medical University, Harbin 150081, China

*** Correspondence:**
Qiuyan Guo

guoqiuyan321@126.com

Hongyu Kuang

ydyneifenmi@163.com

Peng Wang
wpgqy@hrbmu.edu.cn

†The authors wish it to be known that, in their opinion, the first three authors should be regarded as joint First Authors.

1 Supplementary method

1.1 The traits of the participants

The metabolomics data shared in UKB mainly fall into two categories: blood biochemistry and NMR, using the suffixes ". BBC" and ". NMR" to distinguish. Glucose, for example, was measured using two technologies: hexokinase analytical measurements for blood biochemistry (Field ID=30740) and a high-throughput NMR-based metabolic biomarker assay platform developed by Nightingale Health Ltd. Metabolites measured by both types of methods are considered metabolomic traits and are included in the study. The ethnic attributes obtained directly from the UKB include 22 categories (Field ID=21000). These categories were simplified into European, African, Asian, Mixed, and other ethnicities based on region and racial heritage.

1.2 Outcome events

A substantial number of date-type traits were extracted from UKB, which were then utilized to ascertain the most precise right-censored date for the survival status of the participants [Supplementary Table 4]. The utilization of death records (Category ID=100093) constitutes a pivotal element in the determination of the dates of all-cause deaths. Subsequently, the latest recorded date for each participant is considered the right-censored date for all-cause mortality [Supplementary Table 4].

1.3 Statistical analysis

1.3.1 Multi-state model

Based on the multi-state model, to reduce model load and improve stability, a filter was used to remove traits before constructing the multi-state model. The initial procedure entailed the normalization of all continuous traits. A 50-tree random forest model was constructed for the survival data of Transition 1 (Baseline to T2D) and Transition 3 (T2D to Total complication). The following procedure involves the extraction of the traits of the top 10 importance scores of the random forest model [Supplementary Table 6]. The variance inflation factor was calculated to examine the traits with collinearity. The generalized variance inflation factor of all traits is less than 5, and these traits were retained [Supplementary Table 7]. Subsequently, for the continuous traits, they are converted into ordered traits according to the quartiles, with Q1 to Q4 representing the intervals from low to high in sequence. In the multi-state model, the five stages were divided based on the clinical disease progression process of T2D [Supplementary Figures 1A]. The transitions were as follows: [1] Baseline to T2D (n = 1,774); [2] Baseline to death (n = 2,210); [3] T2D to Total complication (n = 798); [4] T2D to death (n = 148); [5] Total complication to death (n = 236). Some sample patients were selected for observation to ascertain the influence of protein levels on the risk estimation of T2D progression. Taking CXCL10 as an example, we selected four patients with protein levels from Q1 to Q4 as examples, while keeping the levels of the other 19 traits at the lowest level. A similar methodology was employed in the selection of sample patients with CCL22

and CCL17 proteins. The cumulative state probability of the sample patients is predicted using the constructed multi-state model [Supplementary Figures 2].

1.3.2 Machine learning methods and statistical methods identifying intersection traits

The traits identified in both machine learning methods and statistical methods were extracted, and their predictive performance was further evaluated. The traits that are identified as significant by both methods are called intersection traits. Specifically, we conducted a ROC analysis to compare the accuracy of intersection traits and common clinical indicators in predicting complications in the test cohort.

1.3.3 Determing the cut-off value for the intersection traits

In addition, a survival analysis of the test cohort was conducted. Specifically, the optimal cut-off values for the continuous intersection traits were determined by maximizing the Youden index. The participants were divided into groups with high and low levels of traits based on the optimal cut-off values [Supplementary Table 17]. KM survival curves were constructed to visually display the clinical progression of complications. The Cox model was used to evaluate the risk value of binary intersection traits. The Cox model was also adjusted for Panel 1[Supplementary Table 18].

1.3.4 Building comprehensive prediction models

After obtaining the intersection traits, we hope to utilize a small number of traits to enhance clinical predictive performance and study the clinical application potential of these traits. The selection of methods was made on the tree structure method (CatBoost, LightGBM, Random Forest and XGBoost), logistic regression, artificial neural networks (multi-layer perceptron and single-layer neural network), Naive Bayes and support vector machine. The intersection traits and clinical indicators were incorporated to construct a comprehensive prediction model. The comprehensive model is then fitted to the training cohort, and the AUC value of the model on the test cohort is calculated. We divided the clinical traits into two groups, Panel 1 and Panel 2. Panel 2 includes some common blood test indicators (such as triglycerides), while Panel 1, in addition to these, also includes some non-blood test indicators (such as sex and smoking) [Supplementary Table 18-19]. To explore the predictive performance of multiple protein or metabolomic trait combinations, the intersection of key traits as a whole was incorporated into the comprehensive model, called full traits. For instance, the full traits that can be utilized for the purpose of predicting DCVD within a timeframe of 5 years encompass the traits including BCAN, ADGRG2, NTPROBNP, RTN4R and GDF15 [Figures 5A]. Similarly, when aiming to predict DKD within a timeframe of 5 years, the full traits are constituted by a collection of traits including COL6A3, NELL1, CAPG, KLK13, IL10, GDF15, GP.Acetyls, CysC and CCN5 [Figures 5B].

2 Supplementary Figures

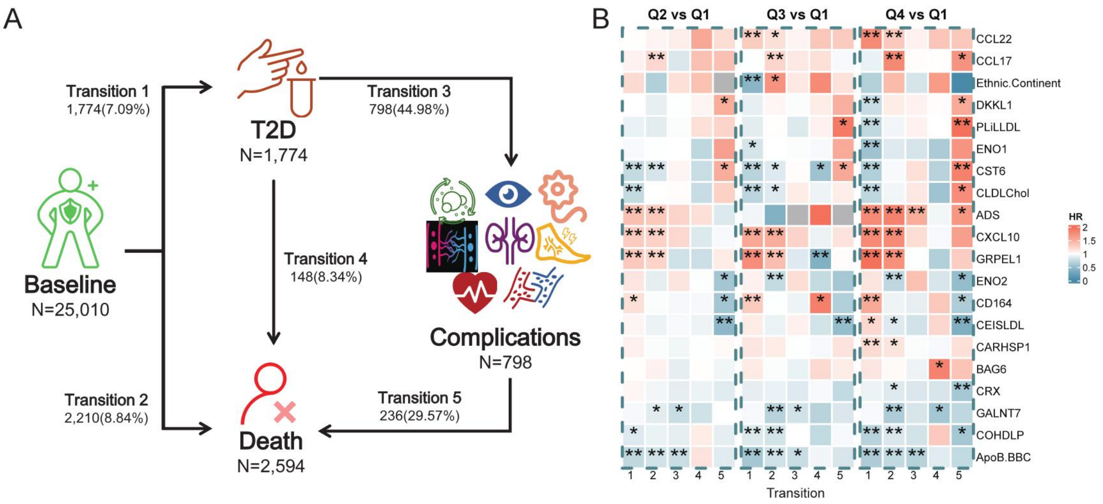

Supplementary Figure 1: The risk of T2D and complications. A. Transitions from baseline to T2D, diabetes complications, and all-cause death. B. Traits affecting the risk of T2D transitions. “*” indicates $p < 0.05$, “**” indicates $p < 0.01$.

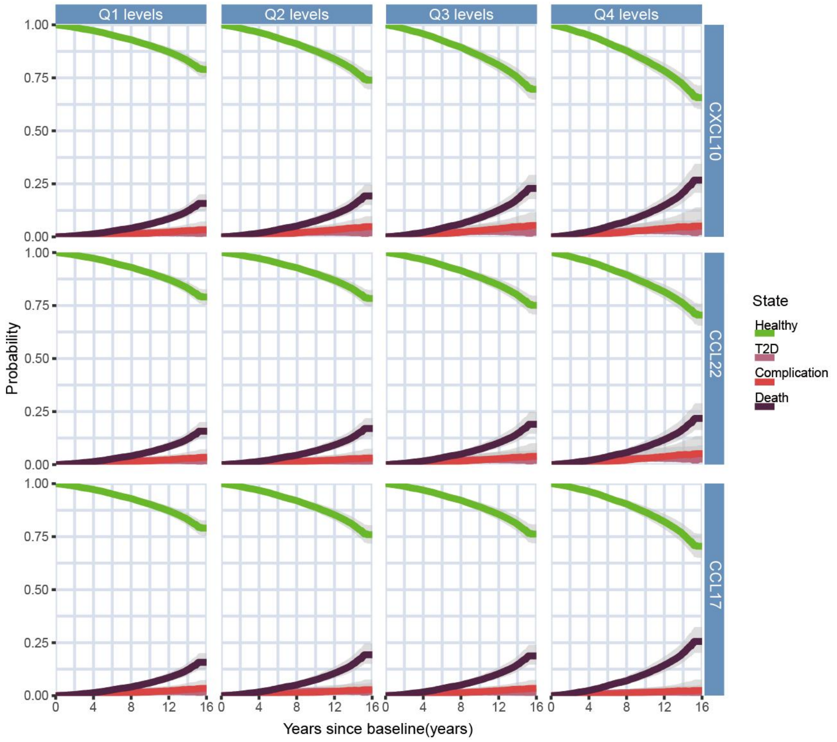

Supplementary Figure 2: Taking CXCL10 as an example, we selected four patients with protein levels from Q1 to Q4 as examples, while keeping the levels of the other 19 traits at the lowest level. A similar methodology was employed in the selection of sample patients for CCL22 and CCL17 proteins. The cumulative state probability of the sample patients is predicted using the constructed multistate model.

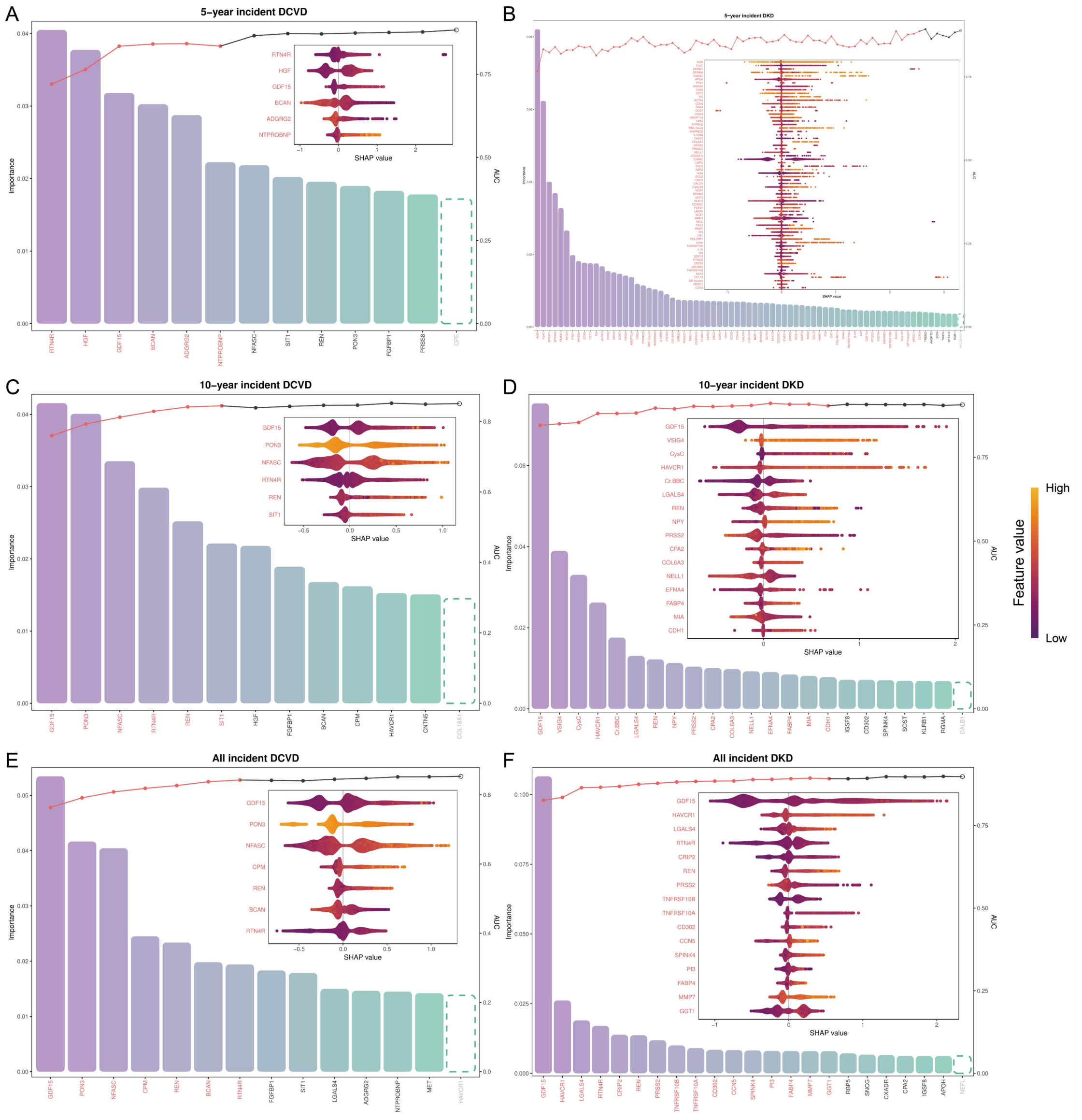

Supplementary Figure 3: The key traits identified by machine learning methods. The bar chart displays the traits assigned the highest importance scores, while the SHAP swarm plot illustrates the contribution of key traits to the prediction of complications. The changes in model AUC resulting from the sequential addition of traits are presented as scatter points and dashed lines. "All incident" indicates an event within 17 years.

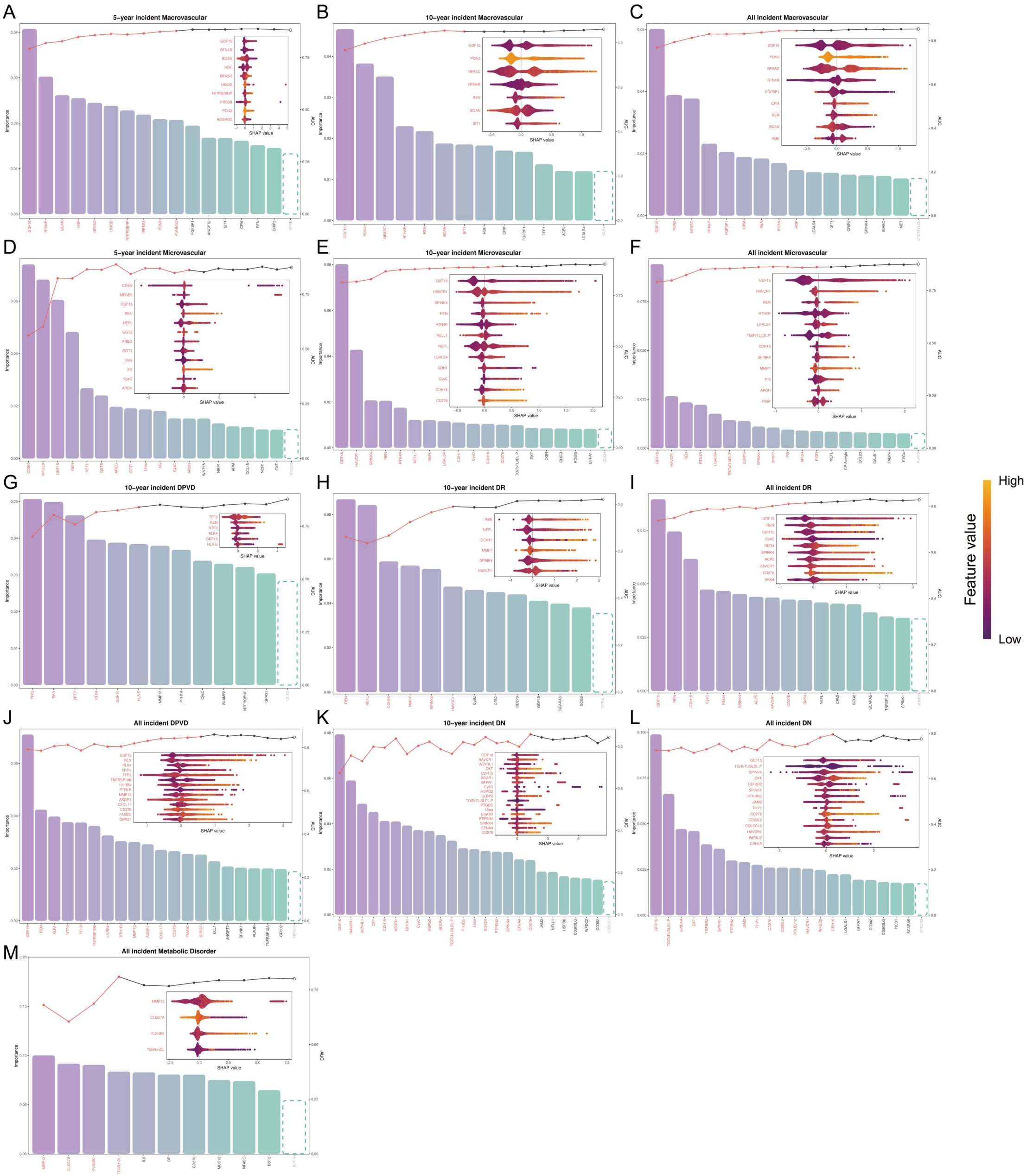

Supplementary Figure 4: The key traits identified by machine learning methods. The bar chart displays the traits assigned the highest importance scores, while the SHAP swarm plot illustrates the contribution of key traits to the prediction of complications. The changes in model AUC resulting from the sequential addition of traits are presented as scatter points and dashed lines. "All incident" indicates an event within 17 years.

A DCVD

Variable	HR(95%CI)	P value	Significance symbol
Protein and Metabolite			
ADGRG2	0.805(0.727-0.891)	0	**
AGRN	0.873(0.774-0.985)	0.027	*
BCAN	0.768(0.677-0.871)	0	**
CD276	1.145(1.035-1.266)	0.008	**
CNTN1	1.165(1.042-1.303)	0.007	**
COL18A1	0.8(0.706-0.906)	0	**
COLEC12	1.227(1.043-1.443)	0.014	*
CPE	0.848(0.779-0.922)	0	**
CPM	1.177(1.049-1.321)	0.006	**
CRHBP	1.112(1.002-1.234)	0.045	*
CTLIMVLDL	0.9(0.83-0.976)	0.011	*
GDF15	1.22(1.103-1.349)	0	**
HAVCR1	1.143(1.036-1.26)	0.008	**
IGFBP4	0.758(0.649-0.886)	0	**
INHBC	1.172(1.047-1.313)	0.006	**
LEPR	0.899(0.812-0.995)	0.04	*
LGALS1	1.158(1.026-1.308)	0.018	*
MET	0.829(0.75-0.916)	0	**
NELL2	0.798(0.716-0.89)	0	**
NFASC	1.383(1.248-1.532)	0	**
NTPROBNP	1.212(1.114-1.32)	0	**
PCSK9	1.172(1.063-1.292)	0.001	**
PON3	0.885(0.822-0.953)	0.001	**
RBP5	0.79(0.701-0.891)	0	**
RTN4R	1.216(1.103-1.342)	0	**
SCARA5	1.18(1.056-1.319)	0.004	**
SIT1	1.153(1.058-1.257)	0.001	**
SLC39A5	0.892(0.801-0.994)	0.038	*
TFF1	1.186(1.081-1.302)	0	**
TNFRSF14	0.863(0.752-0.991)	0.036	*

B DKD

Variable	HR(95%CI)	P value	Significance symbol
Protein and Metabolite			
AGRP	0.831(0.709-0.974)	0.022	*
CA12	1.173(1.01-1.362)	0.037	*
CAPG	1.214(1.055-1.398)	0.007	**
CCN5	1.239(1.068-1.438)	0.005	**
CD164	1.148(1.032-1.277)	0.011	*
CD302	1.386(1.186-1.621)	0	**
CD99L2	0.768(0.658-0.896)	0.001	**
CKAP4	0.723(0.592-0.882)	0.001	**
COL6A3	1.258(1.022-1.549)	0.031	*
CRIP2	1.221(1.059-1.409)	0.006	**
CXADR	0.717(0.603-0.853)	0	**
CysC	1.3(1.101-1.536)	0.002	**
DTX3	0.734(0.619-0.872)	0	**
GDF15	1.278(1.109-1.474)	0.001	**
GP.Acetyls	1.232(1.082-1.403)	0.002	**
HAVCR1	1.194(1.039-1.372)	0.012	*
IL10	1.127(1.006-1.262)	0.038	*
KLK13	1.153(1.009-1.316)	0.036	*
KLRB1	1.17(1.016-1.347)	0.029	*
LGALS7.LGALS7B	0.815(0.692-0.961)	0.015	*
NEFL	1.328(1.165-1.513)	0	**
NELL1	0.848(0.743-0.968)	0.015	*
PHOSPHO1	1.213(1.041-1.413)	0.013	*
PRELP	1.222(1.05-1.423)	0.01	**
PRSS2	0.82(0.723-0.93)	0.002	**
REN	1.229(1.109-1.361)	0	**
RTN4R	1.249(1.1-1.418)	0.001	**

C DN

Variable	HR(95%CI)	P value	Significance symbol
Protein and Metabolite			
CD276	1.447(1.083-1.934)	0.013	*
CDH15	1.303(1.053-1.612)	0.015	*
CysC	0.717(0.519-0.991)	0.044	*
EFNA4	1.592(1.092-2.321)	0.016	*
GDF15	1.413(1.105-1.807)	0.006	**
HAVCR1	1.417(1.069-1.878)	0.015	*
OXT	1.863(1.381-2.514)	0	**
SPINK4	1.509(1.188-1.916)	0.001	**
TGFBR2	0.603(0.423-0.861)	0.005	**
TGiToTLiSLDL.P	1.208(1.087-1.343)	0	**
Urea	1.301(1.093-1.548)	0.003	**

D DPVD

Variable	HR(95%CI)	P value	Significance symbol
Protein and Metabolite			
ASGR1	1.56(1.249-1.948)	0	**
CD276	1.297(1.032-1.629)	0.026	*
CXCL17	1.254(1.037-1.517)	0.02	*
GPR37	1.317(1.034-1.676)	0.026	*
LAYN	0.704(0.542-0.913)	0.008	**
LILRB4	1.32(1.044-1.669)	0.02	*
MMP12	1.332(1.09-1.628)	0.005	**
NPDC1	0.649(0.499-0.843)	0.001	**
PTH1R	1.233(1.058-1.436)	0.007	**
REN	1.522(1.304-1.778)	0	**

Supplementary Figure 5: The key traits identified by statistical methods. Only statistically significant traits are displayed. "*" indicates $p < 0.05$, "**" indicates $p < 0.01$, "0" indicates $p < 0.001$.

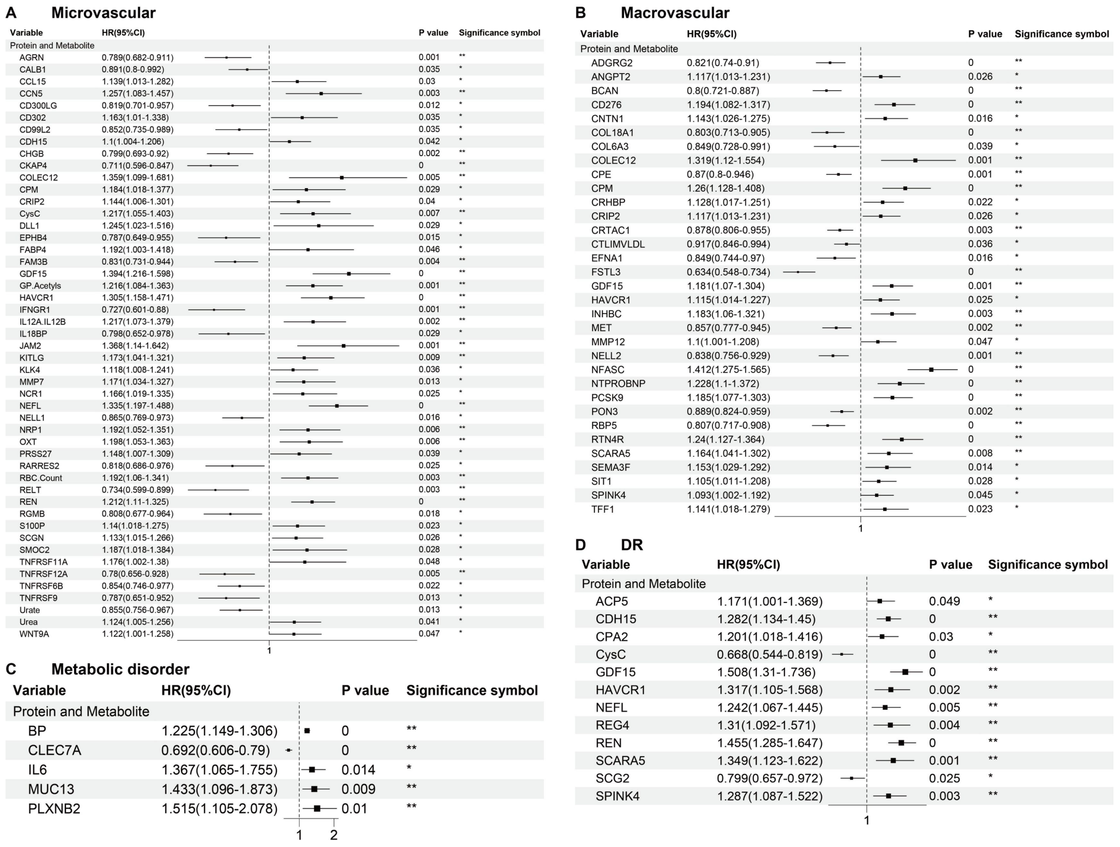

Supplementary Figure 6: The key traits identified by statistical methods. Only statistically significant traits are displayed. A p-value less than 0.05 is indicated by *, and a value less than 0.01 is indicated by **. P-values less than 0.001 are automatically represented as 0 by the program.

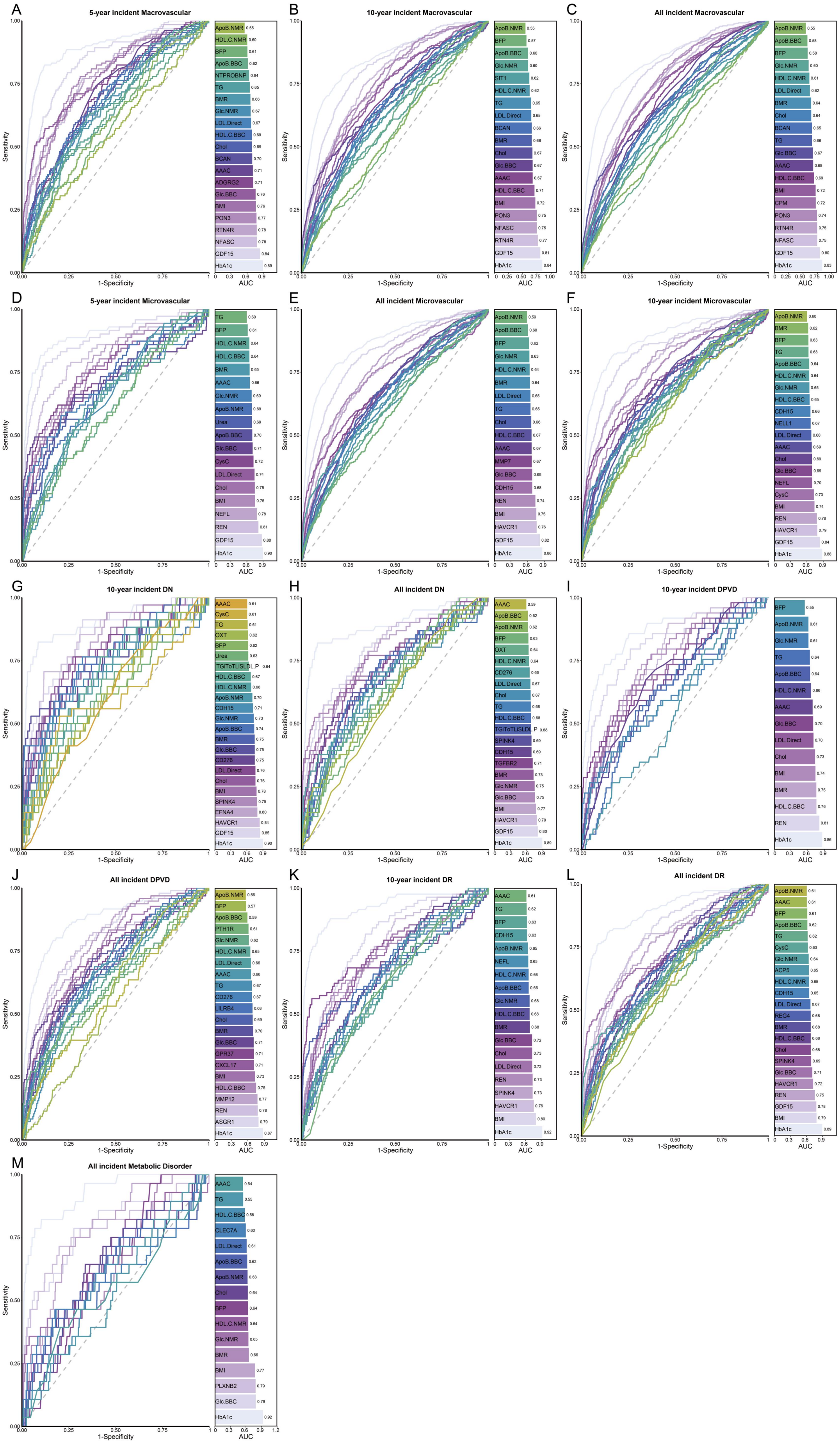
A
5-year incident Macrovascular
B
10-year incident Macrovascular
C
All incident Macrovascular
D
5-year incident Microvascular
E
All incident Microvascular
F
10-year incident Microvascular
G
10-year incident DN
H
All incident DN
I
10-year incident DPVD
J
All incident DPVD
K
10-year incident DR
L
All incident DR
M
All incident Metabolic Disorder
Sensitivity
1-Specificity
AUC

Supplementary Figure 7: The key intersection traits and continuous clinical indicators used to predict future complication events. For each prediction, the continuous clinical indicators remain the same, and the key intersection traits depend on the identification results of the two types of methods.

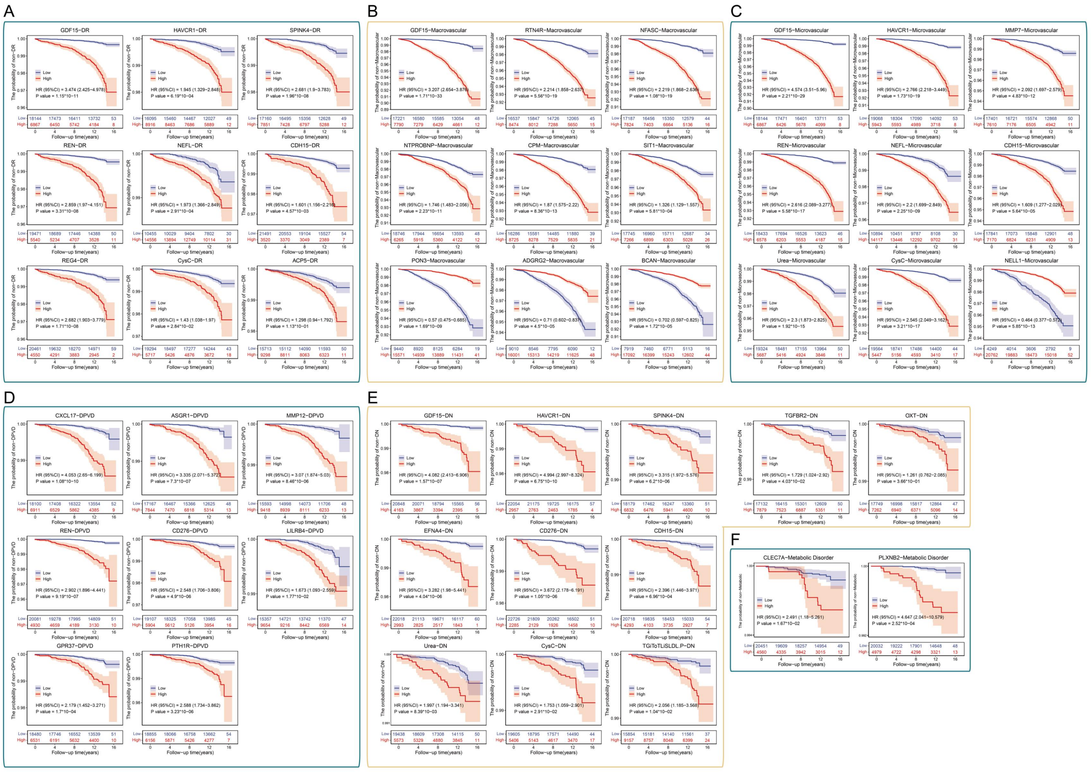

Supplementary Figure 8: The risk differences of binary trait grouping in future complication events. Continuous traits were grouped using the optimal cutoff value, and participants were divided into high and low groups. The future risks of the two groups are presented by the KM curve. The relative risks of the two groups were quantified by the Cox proportional hazards model. The Cox model was also adjusted for the following traits: sex, ethnicity, smoking, alcohol consumption status, insomnia, blood glucose, HbA1c, cholesterol, LDL, triglycerides, HDL-C, apolipoprotein B, age, BMI, basal metabolic rate, and body fat percentage.

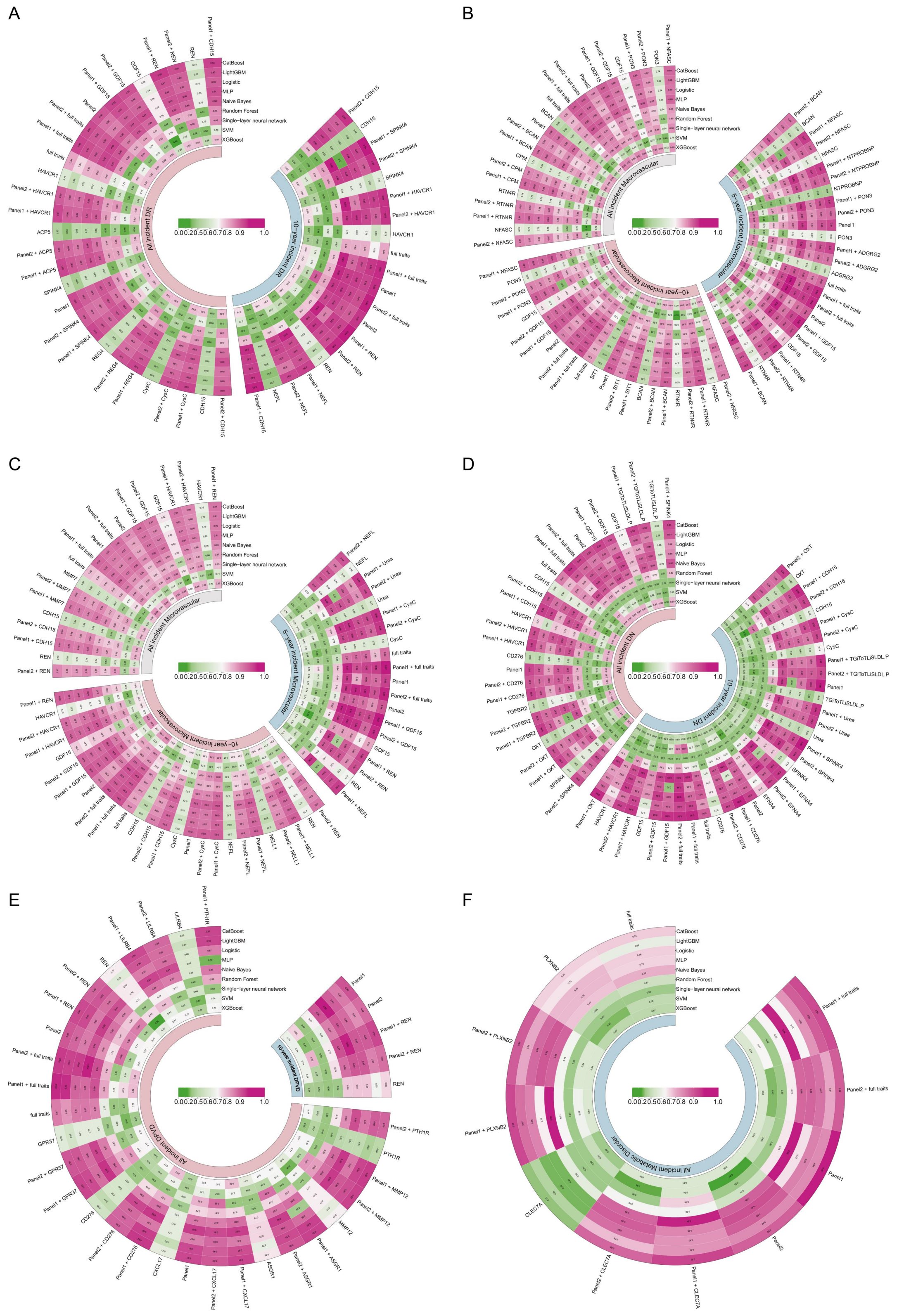

Supplementary Figure 9: The comprehensive model predicting complications.
